# Aging and monocyte immunometabolism in COVID-19

**DOI:** 10.18632/aging.202918

**Published:** 2021-03-30

**Authors:** Brandt D. Pence

**Affiliations:** 1 College of Health Sciences, University of Memphis, Memphis, TN 38152, USA; 2 Center for Nutraceutical and Dietary Supplement Research, University of Memphis, Memphis, TN 38152, USA

**Keywords:** aging, COVID-19, monocytes, immunometabolism, immunosenescence, inflammaging

Severe acute respiratory syndrome coronavirus-2 (SARS-CoV-2) is the third highly pathogenic coronavirus to emerge in the 21^st^ century. The virus, which causes coronavirus disease 2019 (COVID-19), was the first of these coronaviruses to cause a worldwide pandemic, and to date has resulted in over 100 million cases and over 2 million deaths worldwide and has massively impacted the economy of nearly every country. COVID-19 disproportionately affects older adults, with individuals age 80 or older having >20-fold and >300-fold risk of death from COVID-19 compared to 50-59 and 18-39 age groups respectively [1]. Immune dysfunction during aging is well known, and a variety of age-related immune system impairments are thought to impact the response to SARS-CoV-2 in older adults [2]. However, the precise mechanisms by which aging and immunity interact to exacerbate COVID-19 have not been adequately described.

Previously, I suggested a principal role for monocytes in mediating severe COVID-19 in older adults [3]. This argument was predicated on several observations by multiple groups during the early stages of the pandemic. Namely, severe COVID-19 was associated with lung and peripheral tissue infiltration of monocytes and monocyte-derived macrophages, and these cell types also displayed evidence of hyperinflammation and disease-related phenotypic variation which was qualitatively similar to (although more substantial than) changes which occur during the aging process. A number of additional studies have since supported these initial observations. However, molecular mechanisms governing the monocyte response to SARS-CoV-2 are not yet well-characterized, and the interaction between aging and these responses has yet to be described.

In 2018, my laboratory published a short report demonstrating mitochondrial dysfunction in monocytes isolated from older adults [4]. This finding was based on a substantial reduction in maximal respiratory capacity and spare capacity in purified classical monocytes from individuals 60-80 years of age compared to study participants 18-35 years of age. More recently, Saare et al. [5] substantially expanded on our initial work, replicating the observation of mitochondrial dysfunction, and additionally demonstrating increased glucose uptake and altered arachidonic acid metabolism which are indicative of increased basal inflammation in monocytes. These findings support earlier evidence of increased inflammation and cellular dysfunction in aged monocytes [6], suggesting that metabolic changes in the innate immune system related to aging underly (at least in part) the pro-inflammatory state commonly referred to as inflammaging.

Although the molecular basis of the monocyte response to SARS-CoV-2 has yet to be fully described, a few studies have suggested that direct infection of monocytes with the virus initiates metabolic reprogramming indicative of a pro-inflammatory response. Isolated human monocytes respond to SARS-CoV-2 stimulation *in vitro* by upregulating hypoxia-inducible factor (HIF)-1α mediated glycolysis [7], and suppression of this response by pre-treatment of these cells with 2-deoxyglucose (to inhibit glycolysis) or BAY-87-2243 (to inhibit HIF-1α) abrogates the pro-inflammatory and pro-oxidant responses of monocytes to SARS-CoV-2 infection. Likewise, SARS-CoV-2 suppresses mitochondrial respiratory capacity in isolated monocytes [7].

It was also recently observed that monocytes infected with SARS-CoV-2 accumulate intracellular lipid droplets, and that inhibition of this process blocks production of pro-inflammatory cytokines including IL-6, IL-8, and TNFα during viral infection [8]. While the accumulation of intracellular lipids was primarily attributed to increased lipid uptake and triacylglycerol synthesis in infected monocytes, it may also be reflective of impaired fatty acid oxidation by mitochondria in these cells.

Given that SARS-CoV-2 appears to reprogram metabolism in monocytes to promote glucose metabolism and downregulate fatty acid oxidation, it stands to reason that cells which have pre-existing deficits in mitochondrial function (such as monocytes from older individuals) may display exacerbated or aberrant responses to this novel virus. Therefore, age-related metabolic dysfunction in the innate immune system may predispose older individuals to worsened outcomes during COVID-19, contributing to the disproportionate severity of disease in this population. While speculative at this time, these links suggest a therapeutic strategy of immunometabolic modulation may be useful in COVID-19-associated inflammation. Many common geroprotector drugs – including metformin and rapamycin – are also potent regulators of glucose metabolism and therefore have the potential to be repurposed for the treatment of hyperinflammation during COVID-19.
